# Use of anti-viral therapies in hospitalised COVID-19 patients in the United Arab Emirates: a cost-effectiveness and health-care resource use analysis

**DOI:** 10.1186/s12913-023-09376-w

**Published:** 2023-04-20

**Authors:** Ahmad Subhi, Amin Mohamed El Shamy, Saeed Abdullah Mohammed Hussein, James Jarrett, Sam Kozma, Camille Harfouche, Sara Al Dallal

**Affiliations:** 1 Al-Qassimi Hospital Sharjah, Sharjah, United Arab Emirates; 2 Ministry of Health and Prevention, Dubai, United Arab Emirates; 3 Global Medical Solution, Abu Dhabi, United Arab Emirates; 4 Gilead Sciences Inc, London, UK; 5 Gilead Sciences Inc, Dubai, United Arab Emirates; 6 Emirates Health Economics Society, Dubai, United Arab Emirates

**Keywords:** COVID-19, Antivirals, Remdesivir, Healthcare resource use, Cost-effectiveness, United Arab Emirates, Cost

## Abstract

**Background:**

This study attempts to estimate the cost-effectiveness of the antiviral remdesivir, as recommended in the 2021 COVID treatment guidelines for the United Arab Emirates, compared to standard of care (SOC), but also favipiravir (FAVI), which was also recommended for the treatment of hospitalized COVID patients.

**Methods:**

A cost-effectiveness model was built using published efficacy data for RDV, FAVI and SOC as well as local epidemiology data. The outcomes measured included hospital bed days averted, mortality, costs and cost per outcome over one year. One-way, probabilistic and scenario analyses were undertaken to reflect uncertainty in the estimates.

**Results:**

When modelled over one year, the results indicated that treatment of adults in need of supplemental oxygen with RDV + SOC could result in 11,338 fewer general ward bed days, 7,003 fewer ICU days and 5,451 fewer ICU + MIV bed days compared to SOC alone and similar results when compared with FAVI + SOC. The model results also showed that there were 374 fewer deaths associated with the use of RDV + SOC compared to SOC alone. The model also estimates substantial potential cost-savings associated with RDV + SOC treatment compared with SOC alone (USD 3,454 per patient). The results of the one-way sensitivity analysis showed that the model was sensitive to estimates of length of stay and the cost of hospitalization. Despite this, the model predicted cost-savings in all scenarios versus all comparators.

**Conclusions:**

The model estimated that using RDV + SOC could result in substantial reductions in HCRU and cost savings regardless of the comparator. However, it should be noted that reliable clinical information on FAVI was limited therefore it is challenging to interpret these results. All the potential benefits modelled here for RDV + SOC can have implications not only for the health of the UAE population but for improving hospital capacity to deal with other conditions.

**Supplementary Information:**

The online version contains supplementary material available at 10.1186/s12913-023-09376-w.

## Introduction

Severe Acute Respiratory Syndrome Corona Virus type 2 (SARS-CoV-2) emerged at the end of 2019 as a novel coronavirus disease (COVID-19) which rapidly spread worldwide. As of the end of January 2022, there were an estimated 378 million cases of COVID-19 globally, with nearly six million deaths reported [1]. In the United Arab Emirates (UAE), the virus was first detected in March 2020, and there have been approximately 847,142 cases since the beginning of the pandemic, with about 2,248 deaths (January 2022) [2]. When assessed against the priority areas of strategic preparedness issued by the WHO, the UAE promptly deployed risk communication and public engagement, applied contact tracing and management and set up multiple field hospitals to ease the pressure on city-based hospitals and to aid faster response to COVID-19 [3]. In addition, the number of tests conducted per 1000 people in the UAE is one of the highest worldwide [3].

Patients infected with COVID-19 can experience various symptoms, some of which can lead to a substantial clinical burden and premature mortality. Disease progression can occur rapidly and in critical cases can result in mechanical ventilation (MV). Various treatments were developed to fight COVID, including anti-viral therapies and monoclonal antibodies. Early in the pandemic, one of the few approved therapies to treat moderate to severe hospitalized COVID-19 patients was remdesivir (RDV), an antiviral therapy which inhibits the viral RNA-dependent RNA polymerase (RdRp), essential for viral replication [4]. RDV (marketed as Veklury®) received emergency use authorization in May 2020 from the FDA, with full approval in adults in children over 12 years of age in October 2020 [5]. In Europe, RDV received conditional marketing authorization in July 2020 from the EMA and full approval in December 2021 [6]. In the UAE, RDV was granted full regulatory approval in June 2021 [7]. The regulatory approvals were primarily based on the pivotal ACTT-1 trial [4], which compared RDV plus standard of care (SOC) versus placebo plus SOC. The ACTT-1 trial results showed that treatment with remdesivir leads to a shorter time to recovery (15 vs. 10 days) with an increased recovery rate of 29%. In a post-hoc analysis, RDV was associated with a 70% reduction in mortality for patients who required low-flow oxygen support. The findings of the ACCT-1 trial have been supported by similar results from other trials [8] and in real-world comparative effectiveness studies [9, 10]. RDV has also shown continued activity against SARS-COV-2 variants [11]. Favipiravir (FAVI) is another antiviral which inhibits RdRp which at the time of writing (Feb 2022) was being tested against SARS-COV-2 in clinical trials, and several smaller studies had concluded mixed results as to FAVI effectiveness [12–15]. No evidence was identified for the efficacy of FAVI on different variants.

As the pandemic develops and new evidence emerges, guidelines are constantly reviewed and revised. In the 2021 treatment guidelines from the UAE, treatment for hospitalized patients with COVID-19 is based on oxygenation status and risk of progression [16]. For patients with pneumonia or severe pneumonia who require supplemental oxygen, RDV or FAVI is recommended alongside standard of care (SOC)(i.e. dexamethasone or equivalent corticosteroid and other symptom-alleviation related drugs).

Given the strain that COVID-19 care can put on the health care system, it is important to ensure patients are receiving treatments that are delivering good value for money. This study is aims to investigate the cost-effectiveness of RDV + SOC for COVID-19 patients receiving low-flow oxygen in the UAE in line with clinical practice.

## Methods

**Fig. 1:**
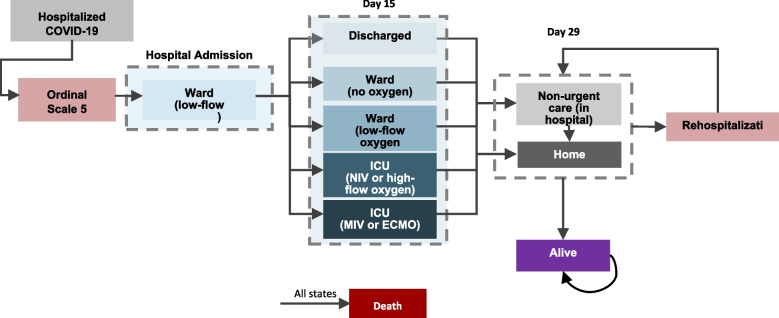
Model Schematic

A health economic model (see Fig. 1) was developed in Microsoft Excel 2016 and conformed to the International Society for Pharmacoeconomic and outcomes Research (ISPOR) Modelling Good Practice Guidelines [17]. The model adopts a health system perspective. As SARS-COV-2 is an acute disease, and data on long-covid impacts are only just emerging, the model assumes a relatively short time horizon of one year. The model cycle length was two weeks, which reflects the primary clinical data as well as real-world information on hospital stays, with a majority of patients leaving hospital (or dying) within a 2-week period and most patients recovering or resolving by one month [18]. For this acute phase, the model adopts a decision tree approach, followed by a Markov model for the remaining time horizon, with cycle length continuing to be two weeks.

A cohort of patients was modelled via a decision tree for the patient’s potential time in hospital (time horizon 29 days) followed by a Markov model for the remaining time horizon (1 year). On day 15, patients transition into one of the following health states based upon their baseline ordinal score (OS) as defined in the ACTT-1 trial, with a minor modifications to reflect local clinical practice [4]: discharged (OS 1–3 plus a negative test), OS 1–3 – awaiting negative test, general ward with no supplemental oxygen (OS4), general ward with low-flow oxygen (OS5), ICU with non-invasive ventilation (NIV) (OS6), ICU with mechanical ventilation (MV) (OS7) or death (OS8). On day 29, patients have either died or assumed to have been discharged and are alive. After day 29, patients enter a Markov model, subject to age- and sex-adjusted mortality derived from UAE-specific lifetables [19]. Patients who are alive can be re-hospitalized post-discharge incurring hospital costs and outcomes associated with OS5 in either the decision tree or the Markov portion of the model.

The model was designed to estimate the number of deaths avoided, hospital days avoided (by ward, ICU and ICU + MV) as well as the associated costs for each arm. Long-term effects of COVID-19 are not considered as part of this model due to a lack of information on the impact of treatment. The model discounts both costs and outcomes at 3.5%, however, as the time horizon is one year, this is not applicable for this analysis. Half-cycle correction is also unnecessary as benefits and costs are almost entirely accrued within the first month with the exception of the natural background mortality in the post-acute Markov cycles which is equal for both arms.

### Population inputs

The population considered in the model base case is calculated using national epidemiological data for 2020 [19]. A hospitalization rate of 10% was applied to the total number of confirmed COVID-19 cases and of those hospitalized, it was assumed that 20% of these would require low-flow oxygen support based on local clinical expert opinion (AS, SAD, AMES, SAMH) [20]. Scenario analyses were performed to investigate potential epidemiological uncertainty, testing a 50% reduction/increase in the number of patients hospitalized who require low-flow oxygen support. An additional scenario was considered including patients who require high-flow oxygen support. In this scenario, it was assumed that 30% of patients would require high-flow oxygen support of the cohort included in the model, and the rest would require low-flow oxygen support based on expert opinion [20]. The average age and gender split were taken from national statistics [21].

### Effectiveness inputs

To populate the model efficacy parameters a literature review was performed in July 2021 to identify relevant clinical trials and real-world evidence for both RDV and FAVI. For RDV, data from the ACTT-1 pivotal phase 3 clinical trial was used in the base-case [4] to compare RDV + SOC and SOC alone. The ACTT-1 study results showed that treatment with RDV + SOC led to a shorter time to recovery (15 days to 10 days, RR: 1.29 (1.12–1.49), *p* < 0.0001) in the overall population. Non-mechanically ventilated patients treated with RDV had less risk of progression to mechanical ventilation or death compared to placebo. In the low-flow oxygen group, to post-hoc analysis, RDV was associated with a 70% significant reduction in mortality among patients requiring low-flow oxygen support (HR: 0.30, 95% CI 0.14–0.64). In addition, analyses were run using a network meta-analysis [22] and two real-world comparative effectiveness studies [9, 10] which compared RDV + SOC vs SOC alone in terms of mortality and time to recovery.

For FAVI, three clinical studies were identified included at least one of the relevant outcomes (time to recovery or discharge and mortality) [13–15]. After a full full-text review, only the Bosaeed et al. study had the relevant patient population (moderate to severe) and outcomes. Therefore, it was chosen to serve as the base-case data for comparing FAVI + SOC and SOC alone [15]. The study was a multi-centre, open-label RCT comparing SOC to FAVI + hydroxychloroquine in moderate to severe patients with COVID-19 in Saudi Arabia which reported non-significant mortality outcomes (6.4% vs 3.8% at day 15, *p* = 0.68; 7.6% vs 10.3% at day 28, *p* = 0.45) and a non-significant increase in the need for ICU or MV (26.4% vs 20.16%, *p* = 0.24; 16.8% vs 15.5%, *p* = 0.78). No published studies comparing RDV and FAVI were identified, therefore the model used a direct comparison for the RDV + SOC vs FAVI + SOC comparison using the point estimates for mortality.

The transition probabilities for RDV + SOC and SOC alone were calculated from Table 1 in Beigel et al. [4] which provided a breakdown of participants by OS and their respective improvements by day 15 (see Supplemental Table 1). As the outcome for disease progression in Bosaeed et al. does not specify OS and was less favourable for FAVI + SOC, it was assumed to have the same transition probability as SOC alone [15].

On day 29, the proportion of patients alive is determined by OS-specific hazard ratios (see Supplemental Table 2) derived from Beigel et al. [4] and Bosaeed et al. [15]. The network meta-analysis and real-world sources are also provided. Given the relatively low death rate in the UAE, a scenario analysis was run by adjusting the transition probabilities to reflect a lower rate of transition (-75%) to death. The additional alive patients are distributed to the other health states using a weighted average of the proportion of patients in each health state in the previous cycle. It is assumed that patients in OS 1–3 at day 29 are exposed to background mortality rates and no treatment effect is applied to these patients.

### Cost and healthcare resource use inputs

The model considered direct costs to the health care setting, including drug costs and inpatient stays (Table 1). The cost of RDV and the cost of FAVI was taken from the government price list of controlled drugs [23]. The cost per hospital day by disease severity was obtained from a the General Circular of the Emirate of Dubai price list [24] which provided the detailed cost per day information including health care professional time, tests and monitoring, and hotel costs. All costs have been converted to USD using the exchange rate of 1 AED = 0.27 USD.

**Table 1:** Cost and length of stay variables

Cost Inputs		Estimate (USD)		Source
RDV per treatment course (6 vials ^a^ USD 499.32 per vial)		2995.92		[23]
FAVI per treatment course (52 200 mg tablets ^a^ USD 2.27 per tablet)		118.04		[23]
OS 1–3 per diem		157.64		[24]
OS 4 per diem		239.31		
OS 5 per diem		807.24		
OS 6 per diem		1437.52		
OS 7 per diem		2708.96		
Readmission (assumed cost per OS4)		239.31		Assumption
**Length of Stay Inputs**				
Score at day 15	General Ward	ICU	ICU + MV	
OS 1–3	2.75			[20]
OS 4	5	0	0	[20]
OS 5	7	0	0	[20]
OS 6	6.75	4.5	0	[20]
OS 7	2	3.5	9.5	[20]
OS 8^a^	1	2	12	[20]
Readmission	5	0	0	[20]

^a^Note that the LOS for OS8 reflects the HCRU associated with patients who end up dying. LOS data was elicited from the clinical experts

Length of stay for each health state (Table 1) was derived from the expert opinion local experts [20]. Patients cumulatively accrued days as per their health state. For example, if a patient transferred from OS5 to OS4 at day 15, by day 29, they will have accrued the hospital days (and costs) associated with both OS5 and OS4. The rate of rehospitalization was also elicited from the four experts.

### Sensitivity and scenario analyses

The model estimated parameter uncertainty in both one-way and probabilistic sensitivity analyses according to the ISPOR best practice guidelines [25] (see supplemental material for variable list). As part of the face-validation of the model, several scenario analyses were created in discussion with the study team, including different efficacy data sources (NMA, RWD), different epidemiological scenarios (± 50% incident cases), UAE-specific mortality reduction scenarios.

## Results

The base-case cohort contained 4,156 patients, with a mean age of 38.4 and 69% of the cohort was male. In the base case, the model estimated 158 deaths in the RDV + SOC arm, compared to 532 and 513 deaths in the SOC alone and FAVI + SOC arms. This means that the model estimated an incremental difference of 374 deaths compared to SOC alone and 355 deaths compared to FAVI + SOC.

The model estimated that using RDV + SOC avoided a total of 11,338 general ward days, 7,003 ICU days, and 5,451 ICU + MV days when compared to SOC alone (or FAVI + SOC) (Table 2). This equates to a reduction of approximately six days hospital stay per patient compared to SOC alone and FAVI + SOC. The model estimated that treatment with FAVI + SOC resulted in 19 fewer deaths than SOC alone. In this comparison, no hospital day reductions were seen as FAVI + SOC efficacy in reducing progression was assumed to equal SOC given the lack of clinical data.

**Table 2:** Estimated outcomes and costs by treatment

Outcomes	Per Patient			Per Cohort			
	Hospital days avoided			Hospital days avoided			COVID-19 Deaths
	Ward	ICU	MIV	Ward	ICU	MIV	
RDV + SOC	7	1	1	30,883	4,964	3,547	158
Standard of Care	10	3	2	42,220	11,966	8,998	532
FAVI + SOC	10	3	2	42,220	11,966	8,998	513
Difference vs SOC	-3	-2	-1	-11,338	-7,003	-5,451	-374
Difference vs FAVI	-3	-2	-1	-11,338	-7,003	-5,451	-355
Difference Favi vs SOC	0	0	0	0	0	0	-19
Costs (USD)	Per Patient			Per Cohort			
	Treatment Cost	Hospitalization Cost	Total Cost	Treatment Cost	Hospitalization Cost	Total Cost	
RDV + SOC	2,992	8,605	11,597	12,434,625	35,761,509	48,196,134	
SOC	0	14,916	14,916	0	61,992,096	61,992,096	
FAVI + SOC	118	14,921	15,039	489,927	62,012,542	62,502,469	
Difference vs SOC^a^	2,992	-6,311	-3,320	12,434,625	-26,230,587	-13,795,962	
Difference vs FAVI^a^	2,874	-6,316	-3,442	11,944,698	-26,251,033	-14,306,335	
Difference FAVI vs SOC	118	5	123	489,927	20,446	510,374	

^a^Negative numbers indicate a cost savings

The model estimated that the estimated acquisition cost of RDV was approximately USD 12,434,625 and the cost of FAVI was approximately USD 489,927. Due to the reduced length of stay, the difference in hospitalization costs between RDV + SOC and SOC alone were USD -26,230,587 and USD -26,251,033 versus FAVI + SOC. This resulted in a cost-savings associated with RDV + SOC of USD -13,795,962. As most of the savings arose from reducing the number of hospitalization days, the use of FAVI + SOC resulted in a total cost increase of USD 510,374. Table 2 provides an overview of the detailed cost results.

The cost-effectiveness results by outcome measure are provided in Table 3. RDV + SOC was less costly and more effective than SOC alone or FAVI + SOC, RDV + SOC was dominant across outcome measures. FAVI + SOC was more costly but had no effect on hospitalization days and consequently was dominated by SOC alone. FAVI + SOC was marginally more effective than SOC in terms of deaths avoided, and therefore an incremental cost-effectiveness ratio (ICER) of USD 27,529 was estimated. Assuming a cost-effectiveness threshold equivalent to GDP per capita in 2022 (approximately USD 36,000) [26] this could be considered cost-effective.

**Table 3:** Incremental cost-effectiveness results from the base-case and other data sources

	Base-case (ACTT-1) results	Associated costs per outcome avoided^a^	Mean (SD) results from data sources	Associated costs per outcome avoided^a^ (SD)
**RDV + SOC vs SOC alone**				
Total COVID-19 deaths avoided	374	-36,886	362 (15)	-30,642 (9,131)
Total Ward days avoided	11,338	-1,217	8,136 (3,784)	-1,468 (309)
Total ICU days avoided	7,003	-1,970	6,930 (104)	-1,593 (450)
Total ICU + MIV days avoided	5,451	-2,531	5,407 (87)	-2,043 (581)
**RDV + SOC vs FAVI + SOC**				
Total COVID-19 deaths avoided	355	-40,245	343 (15)	-33,791 (9,644)
Total Ward days avoided	11,338	-1,262	8,136 (3,784)	-1,544 (349)
Total ICU days avoided	7,003	-2,043	6,930 (104)	-1,667 (449)
Total ICU + MIV days avoided	5,451	-2,625	5,407 (87)	-2,137 (580)
**FAVI + SOC vs SOC alone**				
Total COVID-19 deaths avoided	19	27,529	19 (NA)	27,529 (NA)
Total Ward days avoided	0	0	0	0
Total ICU days avoided	0	0	0	0
Total ICU + MIV days avoided	0	0	0	0

^a^negative values indicates cost-savings

### Sensitivity and scenario analyses

**Fig. 2:**
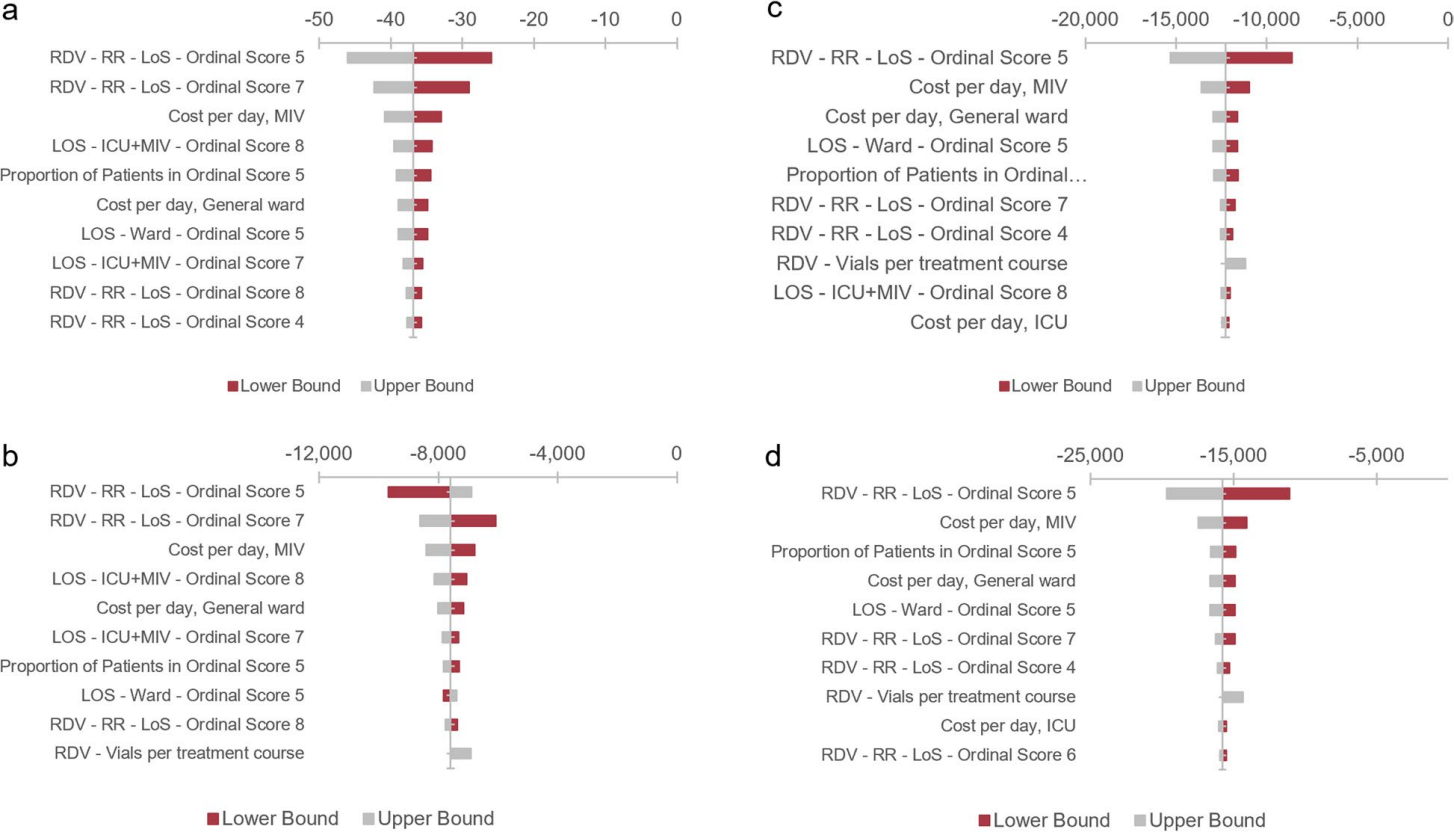
One-way Sensitivity Analysis By Outcome. **a** OWSA for the outcome death avoided; **b** OWSA for the outcome general ward day avoided; **c** OWSA for the outcome ICU day avoided; **d** OWSA for the outcome ICU + MIV day avoided

**Fig. 3:**
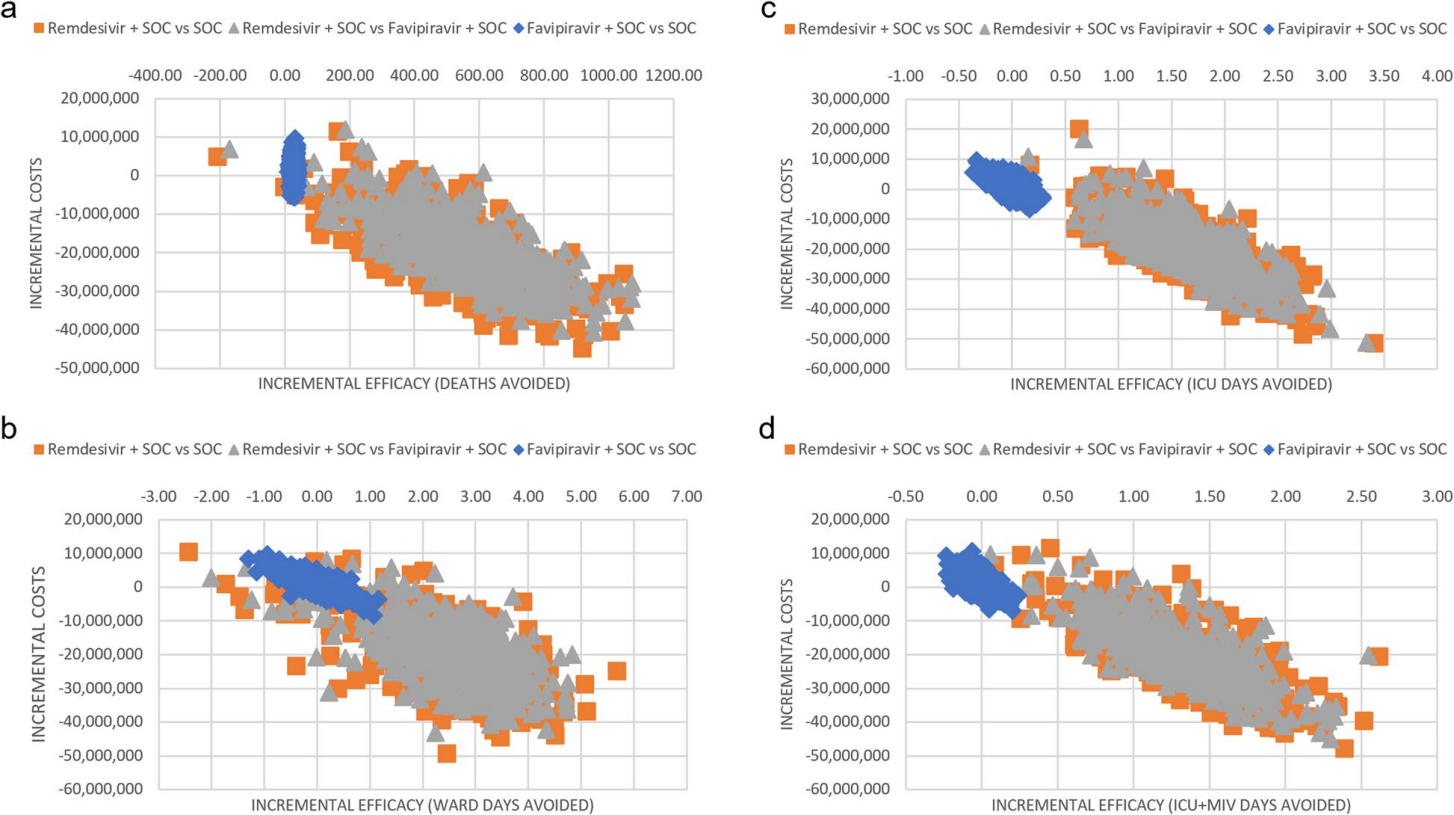
ICER Scatterplot By Outcome. **a** ICER scatterplot for the outcome death avoided; **b** ICER scatterplot for the outcome general ward day avoided; **c** ICER scatterplot for the outcome ICU day avoided; **d** ICER scatterplot for the outcome ICU + MIV day avoided

The results of the one-way sensitivity analysis (see Fig. 2) indicate that the model is sensitive to the relative risk reduction associated with length of stay, as well as the cost per day of hospitalizations. However, regardless of the outcome or scenario, RDV + SOC remains cost-savings compared to SOC alone or FAVI + SOC. The probabilistic sensitivity analysis (see Fig. 3) indicated that RDV + SOC versus SOC alone or FAVI + SOC was the most likely cost-effective option regardless of the outcome and willingness-to-pay threshold, with 98% of iterations resulting in RDV + SOC dominating the other two treatment options (See Supplemental material).

One scenario analysis included varying the source of the efficacy data. Table 3 compares the base case and the mean (SD) result of the three additional data sources. A detailed breakdown of results by data source is available in the supplemental materials. Regardless of the data set or comparator, RDV + SOC was estimated to incrementally reduce deaths and hospital ward days, resulting in subsequent cost-savings.

The results of the epidemiological scenario analysis did not change the overall findings that treatment with RDV + SOC was estimated to result in fewer deaths and hospital days compared to the other two treatment options. However, the scale of the incremental differences changed by the variation in population size (± 50%). The results are presented in Table 4.

**Table 4:** Incremental outcome and cost differences in different scenarios

	Deaths avoided	Ward days avoided	ICU days avoided	ICU + MIV days avoided	Incremental cost difference
Base case					
RDV + SOC vs SOC	-374	-11,338	-7,003	-5,451	-13,795,961.87
RDV + SOC vs FAVI + SOC	-355	-11,338	-7,003	-5,451	-14,306,335.46
FAVI + SOC vs SOC	-19	0	0	0	510,373.59
Cohort size X + 50%					
RDV + SOC vs SOC	-748	-22,678	-14,007	-10,902	-27,595,243.28
RDV + SOC vs FAVI + SOC	-711	-22,678	-14,007	-10,902	-28,616,113.25
FAVI + SOC vs SOC	-37	0	0	0	1,020,869.98
Cohort size X -50%					
RDV + SOC vs SOC	-187	-5,669	-3,501	-2,725	-6,897,980.94
RDV + SOC vs FAVI + SOC	-178	-5,669	-3,501	-2,725	-7,153,167.73
FAVI + SOC vs SOC	-9	0	0	0	255,186.79
Reduction in SOC mortality rate					
RDV + SOC vs SOC	-100	-11,563	-6,753	-4,865	-12,694,191.05
RDV + SOC vs FAVI + SOC	-96	-11,563	-6,753	-4,865	-13,211,503.68
FAVI + SOC vs SOC	-5	0	0	0	517,312.63
Addition of high-flow oxygen patient group (and SOC mortality reduction)					
RDV + SOC vs SOC	-177	-17,866	-11,212	-8,159	-16,535,860.11
RDV + SOC vs FAVI + SOC	-165	-17,866	-11,212	-8,159	-17,582,425.43
FAVI + SOC vs SOC	-13	0	0	0	1,046,565.33

In the base case, the model estimated 532 deaths in the SOC arm. This would account for 80% of the total observed deaths in the UAE in 2020 (*n* = 669), indicating that the model base case was likely overestimating the number of deaths. An adjustment to the transition probabilities was made which reduced the proportion of patients dying at day 15 by 75%. This scenario results in the model predicting 143 deaths in the SOC arm compared to 43 deaths in the RDV + SOC arm (100 fewer than SOC) and 139 deaths in the FAVI + SOC arm (5 fewer than SOC).

As the UAE guidelines recommend using an antiviral in the case of low- and high-flow patients, a scenario was run including a cohort of both groups, assuming the overall cohort includes a total of 40% of the hospitalized patients (*n* = 8,313). It was assumed that when hospitalized, approximately 69% would be low-flow patients and 31% would be high-flow oxygen patients based on the Beigel et al. (2020) paper. As per the base-case, the model over-predicts the number of deaths (*n* = 1,500), therefore this scenario was combined with the reduced mortality scenario described previously. The model results show that RDV + SOC reduces deaths, hospital days, and costs compared to SOC alone or FAVI + SOC.

## Discussion/Conclusions

The model has shown that the treatment of hospitalized COVID-19 who need low-flow oxygen can benefit from anti-viral treatment and that these benefits can lead to substantial health-care savings. The model estimated that treating low-flow oxygen patients with RDV + SOC resulted in areduction in deaths (between 100 and 374 depending on the scenario) as well as hospital bed days regardless of the comparator, which ultimately results in substantial health-care savings (USD 13.8 million) over one-year. These findings were substantiated in all scenarios tested as well as the parameter uncertainty (one-way and probabilistic).

While the model results appear robust, the model is sensitive to the relative risk reduction of length of stay. As length of stay was derived from expert opinion, this is a source of uncertainty. Evidence from the real world on a cohort of patients in the population of interest could reduce this uncertainty. In addition, the data on FAVI + SOC is limited. No head-to-head data exists between FAVI + SOC and RDV + SOC, and trial data in the literature versus SOC shows mixed results. Therefore, any comparisons with this anti-viral should be interpreted with caution. Subsequent to the conduct of this study, FAVI + SOC is no longer recommended by the UAE guidelines after an examination of emerging evidence did not show a benefit over SOC alone.

As part of the validation of model results, it was apparent that the data from the ACTT-1 study had much higher rates of mortality than would be expected to be seen in the UAE based on local epidemiological data. While we have tried to control for this by conducting a scenario analysis, this artificial adjustment can lead to bias in the estimates even while it may better reflect the local population experience. It can also be noted that ignoring the potential mortality benefit entirely would not change the overall findings that the use of RDV + SOC compared to SOC alone can potentially be cost savings as the majority of the modelled benefits arise from preventing progression and reducing the overall time to discharge.

The model does not directly address the different variants associated with SARS-COV-2. However, evidence has shown that RDV remains effective against the different variants so far seen in the pandemic [11] and different real-world analyses have shown that despite differences in hospitalization rates over time, for those in hospital the length of stay and has largely remained similar over time [18], as has the progression for patients who are on low-flow oxygen. This indicates that the results of this model may be valid despite the different variants over time. Finally, the model does not consider the long-term consequences of COVID-19, which could result in an underestimation of the benefit of early anti-viral treatment. This is an area for future research.

This model has estimated the potential for substantial clinical and economic benefit in treating COVID-19 patients who require low-flow oxygen support across a wide variety of scenarios in the UAE. While the COVID-19 situation in the UAE has seen a relatively low incidence of hospitalizations and deaths compared to the rest of the world, during the peaks of the pandemics, hospital beds were at a premium. As new therapies emerge in an outpatient setting, such as the recent extension of the label for RDV [27] and nirmatrelvir + ritonavir [28], both of which saw significant reductions of hospitalization or death (87% and 89% respectively), there is a chance that earlier use of antivirals could keep many people out of hospital entirely. However, only RDV is currently used in patients who are hospitalized in the UAE. It should be noted that nirmatrelvir + ritonavir has an ongoing clinical trial in the inpatient setting. Therefore, there is likely a need for a reassessment of the cost-effectiveness of antiviral therapies for treatment of COVID-19 in the future.

As the COVID-19 pandemic continues, it is essential to have a wide variety of potential treatments that can reduce the burden of the disease on patients and the health care system. As new treatments emerge for both the outpatient and in-patient setting, it is essential to understand the best approach to maximise patient outcomes and reduce the health care system burden.

## Supplementary Information

**Additional file 1: Table S1.** Transition probabilities at day 15. **Table S2.** Day 28 Mortality Hazard Ratios. **Table S3.** Results by efficacy data source. **Table S4.** Parameters included in the sensitivity analyses.

## Data Availability

Data used in this model is publicly available and the references are provided in the reference list with the exception of the network meta-analysis which is currently under peer review. This can be requested from the corresponding author.
