# Supplementary material for: Use of anti-viral therapies in hospitalised COVID-19 patients in the United Arab Emirates: a cost-effectiveness and health-care resource use analysis
Source: BMC Health Serv Res. 2023 Apr 20;23:383. doi: 10.1186/s12913-023-09376-w (PMC10116096; doi:10.1186/s12913-023-09376-w)
Supplement: Supplementary file 1 — Additional file 1: Table S1. Transition probabilities at day 15. Table S2. Day 28 Mortality Hazard Ratios. Table S3. Results by efficacy data source. Table S4. Parameters included in the sensitivity analyses. [file 12913_2023_9376_MOESM1_ESM.docx]

Supplementary appendix

Table S1: Transition probabilities at day 15

| OS at Baseline | Ordinal Score 4 | | Ordinal Score 5 | | Ordinal Score 6 | | Ordinal Score 7 | |
| --- | --- | --- | --- | --- | --- | --- | --- | --- |
| OS at day 15 | SoC/FAVI | RDV | SoC/FAVI | RDV | SoC/FAVI | RDV | SoC/FAVI | RDV |
| OS 1-3 | 75% | 88% | 61% | 72% | 34% | 42% | 14% | 12% |
| OS 4 | 11% | 4% | 6% | 7% | 4% | 13% | 6% | 5% |
| OS 5 | 8% | 4% | 9% | 11% | 14% | 2% | 15% | 21% |
| OS 6 | 0% | 1% | 3% | 2% | 11% | 13% | 4% | 8% |
| OS 7 | 5% | 1% | 10% | 6% | 20% | 17% | 48% | 44% |
| OS 8 | 2% | 1% | 10% | 3% | 16% | 14% | 14% | 11% |

OS: Ordinal Score; FAVI: favipiravir+standard of care; RDV: remdesivir + standard of care; SoC: standard of care

Table S2: Day 28 Mortality Hazard Ratios

|  | Hazard Ratio - Source Data | | | | |
| --- | --- | --- | --- | --- | --- |
| RDV | ACTT-1 (95% CI) | NMA (95% CI) | Chokkalingham (95% CI) | Mozaffari (95% CI) | Bosaeed* (95% CI) |
| OS 1-3 | 1 | 1 | 1 | 1 | 1 |
| OS 4 | 0.42 (0.04,0.67) | 0.42 (0.04,0.67) | 0.87 (0.80,0.94) | 0.80 (0.68, 0.94) | 0.71 (0.10, 1.33) |
| OS 5 | 0.28 (0.12, 0.66) | 0.24 (0.11,0.48) | 0.78 (0.69, 0.87) | 0.77 (0.68, 0.86) | 0.71 (0.10, 1.33) |
| OS 6 | 0.82 (0.40, 1.69) | 0.9 (0.51,1.56) | 0.73 (0.66, 0.80) | 0.97 (0.84, 1.11) | 0.71 (0.10, 1.33) |
| OS 7 | 0.76 (0.39,1.50) | 0.76 (0.39,1.50) | 0.76 (0.66, 0.88) | 0.81 (0.69, 0.94) | 0.71 (0.10, 1.33) |

*Bosaeed data calculated from values provided in Table 2. CI: confidence interval; RDV: remdesivir+standard of care; OS: Ordinal Score;

Table S3: Results by efficacy data source

|  | ACTT-1 | NMA | Chokkalingham  et al | Mozaffari  et al | Mean | Min | Max | SD |
| --- | --- | --- | --- | --- | --- | --- | --- | --- |
| **RDV + SOC vs SOC alone** | | | | | | | | |
| **Total COVID-19 deaths avoided** | 374 | 375 | 351 | 347 | 362 | 347 | 375 | 15 |
| **Total Ward days avoided** | 11,338 | 5,916 | 3,951 | 11,338 | 8,136 | 3,951 | 11,338 | 3,784 |
| **Total ICU days avoided** | 7,003 | 6,934 | 6,781 | 7,003 | 6,930 | 6,781 | 7,003 | 104 |
| **Total ICU + MIV days avoided** | 5,451 | 5,451 | 5,277 | 5,451 | 5,407 | 5,277 | 5,451 | 87 |
|  |  |  |  |  |  |  |  |  |
| **Savings per death avoided** | -36,886 | -25,105 | -20,796 | -39,783 | -30,642 | -39,783 | -20,796 | 9,131 |
| **Savings per ward day avoided** | -1,217 | -1,590 | -1,848 | -1,217 | -1,468 | -1,848 | -1,217 | 309 |
| **Savings per ICU day avoided** | -1,970 | -1,356 | -1,077 | -1,970 | -1,593 | -1,970 | -1,077 | 450 |
| **Savings per ICU + MIV day avoided** | -2,531 | -1,725 | -1,384 | -2,531 | -2,043 | -2,531 | -1,384 | 581 |
|  | | | | | | | | |
| **RDV + SOC vs FAVI + SOC** | | | | | | | | |
| **Total COVID-19 deaths avoided** | 355 | 356 | 333 | 328 | 343 | 328 | 356 | 15 |
| **Total Ward days avoided** | 11,338 | 5,916 | 3,951 | 11,338 | 8,136 | 3,951 | 11,338 | 3,784 |
| **Total ICU days avoided** | 7,003 | 6,934 | 6,781 | 7,003 | 6,930 | 6,781 | 7,003 | 104 |
| **Total ICU + MIV days avoided** | 5,451 | 5,451 | 5,277 | 5,451 | 5,407 | 5,277 | 5,451 | 87 |
|  |  |  |  |  |  |  |  |  |
| **Savings per death avoided** | -40,245 | -27,846 | -23,490 | -43,585 | -33,791 | -43,585 | -23,490 | 9,644 |
| **Savings per ward day avoided** | -1,262 | -1,676 | -1,977 | -1,262 | -1,544 | -1,977 | -1,262 | 349 |
| **Savings per ICU day avoided** | -2,043 | -1,430 | -1,152 | -2,043 | -1,667 | -2,043 | -1,152 | 449 |
| **Savings per ICU + MIV day avoided** | -2,625 | -1,819 | -1,480 | -2,625 | -2,137 | -2,625 | -1,480 | 580 |
|  | | | | | | | | |
| **FAVI + SOC vs SOC alone*** | | | | | | | | |
| **Total COVID-19 deaths avoided** | 19 | | | | | | | |
| **Costs per death avoided** | 96,152 | | | | | | | |

FAVI: favipiravir + standard of care; ICU: Intensive Care Unit; MIV: mechanical ventilation; NMA: network meta analysis; OS: Ordinal Score; RDV: remdesivir+standard of care; SoC: standard of care

Table S4: Parameters included in the sensitivity analyses

| Parameter Name | Value | Distribution | SE | Alpha | Beta |
| --- | --- | --- | --- | --- | --- |
| Disutility - General Ward | 0.27 | Beta | 0.013776 | 280.1668 | 757.488 |
| Disutility - ICU | 0.36 | Beta | 0.018367 | 245.5024 | 436.4487 |
| Disutility - ICU + MIV | 0.56 | Beta | 0.028571 | 168.4704 | 132.3696 |
| Disutility - Rehabilitation | 0.01 | Beta | 0.00051 | 380.3084 | 37650.53 |
| Disutility - Rehospitalization | 0.002959 | Beta | 0.000151 | 383.0203 | 129063.7 |
| Disutility - Acute Kidney Injury | 0.11 | Beta | 0.005612 | 341.7924 | 2765.411 |
| Disutility - Pulmonary Fibrosis | 0.1 | Beta | 0.005102 | 345.644 | 3110.796 |
| RDV - Cost per Vial | 1834 | Gamma | 25.47551 | 384.16 | 1.299771 |
| RDV - Vials per treatment course | 6 | Gamma | 0.089286 | 4515.84 | 0.001329 |
| Comparator treatment cost | 432.9 | Gamma | 6.022449 | 384.16 | 0.307268 |
| Cost per day OS 1-3 | 579 | Gamma | 29.54082 | 384.16 | 1.507185 |
| Cost per day OS4 | 879 | Gamma | 44.84694 | 384.16 | 2.288109 |
| Cost per day, General ward | 2965 | Gamma | 41.18571 | 384.16 | 2.101312 |
| Cost per day, ICU | 5280 | Gamma | 73.34286 | 384.16 | 3.741983 |
| Cost per day, MIV | 9950 | Gamma | 138.2122 | 384.16 | 7.051645 |
| Cost per day of rehabilitation post-discharge | 579 | Gamma | 8.042857 | 384.16 | 0.41035 |
| Duration of rehabilitations (days) | 5 | Gamma | 0.255102 | 384.16 | 0.013015 |
| Cost of hospital readmission | 879 | Gamma | 12.20969 | 384.16 | 0.622944 |
| Productivity loss cost per day | 268.15 | Gamma | 13.68112 | 384.16 | 0.698016 |
| Proportion of Patients in Ordinal Score 5 | 0.692675 | Beta | 0.05102 | -1 | 0 |
| SoC - Day 15 - Baseline Ordinal Score 4 - Ordinal Score 1-3 | 0.746032 | Beta | 0.038063 | 96.81841 | 32.95946 |
| SoC - Day 15 - Baseline Ordinal Score 4 - Ordinal Score 4 | 0.111111 | Beta | 0.005669 | 341.3644 | 2730.916 |
| SoC - Day 15 - Baseline Ordinal Score 4 - Ordinal Score 5 | 0.079365 | Beta | 0.004049 | 353.5917 | 4101.664 |
| SoC - Day 15 - Baseline Ordinal Score 4 - Ordinal Score 6 | 0 | Beta | 0 | 0 | 0 |
| SoC - Day 15 - Baseline Ordinal Score 4 - Ordinal Score 7 | 0.047619 | Beta | 0.00243 | 365.819 | 7316.381 |
| SoC - Day 15 - Baseline Ordinal Score 5 - Ordinal Score 1-3 | 0.610837 | Beta | 0.031165 | 148.8899 | 94.85724 |
| SoC - Day 15 - Baseline Ordinal Score 5 - Ordinal Score 4 | 0.064039 | Beta | 0.003267 | 359.4946 | 5254.152 |
| SoC - Day 15 - Baseline Ordinal Score 5 - Ordinal Score 5 | 0.08867 | Beta | 0.004524 | 350.0079 | 3597.303 |
| SoC - Day 15 - Baseline Ordinal Score 5 - Ordinal Score 6 | 0.034483 | Beta | 0.001759 | 370.8786 | 10384.6 |
| SoC - Day 15 - Baseline Ordinal Score 5 - Ordinal Score 7 | 0.103448 | Beta | 0.005278 | 344.3159 | 2984.071 |
| SoC - Day 15 - Baseline Ordinal Score 6 - Ordinal Score 1-3 | 0.336735 | Beta | 0.01718 | 254.4633 | 501.2155 |
| SoC - Day 15 - Baseline Ordinal Score 6 - Ordinal Score 4 | 0.040816 | Beta | 0.002082 | 368.4392 | 8658.321 |
| SoC - Day 15 - Baseline Ordinal Score 6 - Ordinal Score 5 | 0.142857 | Beta | 0.007289 | 329.1371 | 1974.823 |
| SoC - Day 15 - Baseline Ordinal Score 6 - Ordinal Score 6 | 0.112245 | Beta | 0.005727 | 340.9278 | 2696.429 |
| SoC - Day 15 - Baseline Ordinal Score 6 - Ordinal Score 7 | 0.204082 | Beta | 0.010412 | 305.5559 | 1191.668 |
| SoC - Day 15 - Baseline Ordinal Score 7 - Ordinal Score 1-3 | 0.136364 | Beta | 0.006957 | 331.6382 | 2100.375 |
| SoC - Day 15 - Baseline Ordinal Score 7 - Ordinal Score 4 | 0.058442 | Beta | 0.002982 | 361.6506 | 5826.594 |
| SoC - Day 15 - Baseline Ordinal Score 7 - Ordinal Score 5 | 0.149351 | Beta | 0.00762 | 326.6361 | 1860.406 |
| SoC - Day 15 - Baseline Ordinal Score 7 - Ordinal Score 6 | 0.038961 | Beta | 0.001988 | 369.1538 | 9105.793 |
| SoC - Day 15 - Baseline Ordinal Score 7 - Ordinal Score 7 | 0.480519 | Beta | 0.024516 | 199.0831 | 215.225 |
| Day 29 Mortality - Ordinal Score 4 | 0.015873 | Beta | 0.00081 | 378.0463 | 23438.87 |
| Day 29 Mortality - Ordinal Score 5 | 0.098522 | Beta | 0.005027 | 346.2132 | 3167.851 |
| Day 29 Mortality - Ordinal Score 6 | 0.163265 | Beta | 0.00833 | 321.2767 | 1646.543 |
| Day 29 Mortality - Ordinal Score 7 | 0.136364 | Beta | 0.006957 | 331.6382 | 2100.375 |
| LOS - Ward - Ordinal Score 1-3 | 2 | Gamma | 0.140306 | 384.16 | 0.007158 |
| LOS - Ward - Ordinal Score 4 | 5 | Gamma | 0.255102 | 384.16 | 0.013015 |
| LOS - Ward - Ordinal Score 5 | 7 | Gamma | 0.357143 | 384.16 | 0.018222 |
| LOS - Ward - Ordinal Score 6 | 5 | Gamma | 0.344388 | 384.16 | 0.017571 |
| LOS - Ward - Ordinal Score 7 | 5 | Gamma | 0.102041 | 384.16 | 0.005206 |
| LOS - Ward - Ordinal Score 8 | 2.5 | Gamma | 0.05102 | 384.16 | 0.002603 |
| LOS - ICU - Ordinal Score 6 | 5 | Gamma | 0.229592 | 384.16 | 0.011714 |
| LOS - ICU - Ordinal Score 7 | 2 | Gamma | 0.178571 | 384.16 | 0.009111 |
| LOS - ICU - Ordinal Score 8 | 2.5 | Gamma | 0.102041 | 384.16 | 0.005206 |
| LOS - ICU+MIV - Ordinal Score 7 | 10 | Gamma | 0.484694 | 384.16 | 0.024729 |
| LOS - ICU+MIV - Ordinal Score 8 | 12 | Gamma | 0.612245 | 384.16 | 0.031237 |
| Proportion of patients rehospitalized | 0.12 | Beta | 0.003954 | 354.3101 | 4217.433 |
| RDV- Day 15 - Baseline Ordinal Score 4 - Ordinal Score 1-3 | 0.88 | Beta | 0.044898 | 45.2192 | 6.166255 |
| RDV- Day 15 - Baseline Ordinal Score 4 - Ordinal Score 4 | 0.04 | Beta | 0.002041 | 368.7536 | 8850.086 |
| RDV- Day 15 - Baseline Ordinal Score 4 - Ordinal Score 5 | 0.04 | Beta | 0.002041 | 368.7536 | 8850.086 |
| RDV- Day 15 - Baseline Ordinal Score 4 - Ordinal Score 6 | 0.013333 | Beta | 0.00068 | 379.0245 | 28047.82 |
| RDV- Day 15 - Baseline Ordinal Score 4 - Ordinal Score 7 | 0.013333 | Beta | 0.00068 | 379.0245 | 28047.82 |
| Comp- Day 15 - Baseline Ordinal Score 4 - Ordinal Score 1-3 | 0.746032 | Beta | 0.038063 | 96.81841 | 32.95946 |
| Comp- Day 15 - Baseline Ordinal Score 4 - Ordinal Score 4 | 0.111111 | Beta | 0.005669 | 341.3644 | 2730.916 |
| Comp- Day 15 - Baseline Ordinal Score 4 - Ordinal Score 5 | 0.079365 | Beta | 0.004049 | 353.5917 | 4101.664 |
| Comp- Day 15 - Baseline Ordinal Score 4 - Ordinal Score 6 | 0 | Beta | 0 | 0 | 0 |
| Comp- Day 15 - Baseline Ordinal Score 4 - Ordinal Score 7 | 0.047619 | Beta | 0.00243 | 365.819 | 7316.381 |
| RDV- Day 15 - Baseline Ordinal Score 5 - Ordinal Score 1-3 | 0.715517 | Beta | 0.036506 | 108.5714 | 43.16693 |
| RDV- Day 15 - Baseline Ordinal Score 5 - Ordinal Score 4 | 0.073276 | Beta | 0.003739 | 355.9371 | 4501.557 |
| RDV- Day 15 - Baseline Ordinal Score 5 - Ordinal Score 5 | 0.107759 | Beta | 0.005498 | 342.6557 | 2837.189 |
| RDV- Day 15 - Baseline Ordinal Score 5 - Ordinal Score 6 | 0.021552 | Beta | 0.0011 | 375.8591 | 17064 |
| RDV- Day 15 - Baseline Ordinal Score 5 - Ordinal Score 7 | 0.056034 | Beta | 0.002859 | 362.5778 | 6108.041 |
| Comp- Day 15 - Baseline Ordinal Score 5 - Ordinal Score 1-3 | 0.610837 | Beta | 0.031165 | 148.8899 | 94.85724 |
| Comp- Day 15 - Baseline Ordinal Score 5 - Ordinal Score 4 | 0.064039 | Beta | 0.003267 | 359.4946 | 5254.152 |
| Comp- Day 15 - Baseline Ordinal Score 5 - Ordinal Score 5 | 0.08867 | Beta | 0.004524 | 350.0079 | 3597.303 |
| Comp- Day 15 - Baseline Ordinal Score 5 - Ordinal Score 6 | 0.034483 | Beta | 0.001759 | 370.8786 | 10384.6 |
| Comp- Day 15 - Baseline Ordinal Score 5 - Ordinal Score 7 | 0.103448 | Beta | 0.005278 | 344.3159 | 2984.071 |
| RDV- Day 15 - Baseline Ordinal Score 6 - Ordinal Score 1-3 | 0.421053 | Beta | 0.021482 | 221.9874 | 305.2326 |
| RDV- Day 15 - Baseline Ordinal Score 6 - Ordinal Score 4 | 0.126316 | Beta | 0.006445 | 335.5082 | 2320.598 |
| RDV- Day 15 - Baseline Ordinal Score 6 - Ordinal Score 5 | 0.021053 | Beta | 0.001074 | 376.0514 | 17486.39 |
| RDV- Day 15 - Baseline Ordinal Score 6 - Ordinal Score 6 | 0.126316 | Beta | 0.006445 | 335.5082 | 2320.598 |
| RDV- Day 15 - Baseline Ordinal Score 6 - Ordinal Score 7 | 0.168421 | Beta | 0.008593 | 319.2909 | 1576.499 |
| Comp- Day 15 - Baseline Ordinal Score 6 - Ordinal Score 1-3 | 0.336735 | Beta | 0.01718 | 254.4633 | 501.2155 |
| Comp- Day 15 - Baseline Ordinal Score 6 - Ordinal Score 4 | 0.040816 | Beta | 0.002082 | 368.4392 | 8658.321 |
| Comp- Day 15 - Baseline Ordinal Score 6 - Ordinal Score 5 | 0.142857 | Beta | 0.007289 | 329.1371 | 1974.823 |
| Comp- Day 15 - Baseline Ordinal Score 6 - Ordinal Score 6 | 0.112245 | Beta | 0.005727 | 340.9278 | 2696.429 |
| Comp- Day 15 - Baseline Ordinal Score 6 - Ordinal Score 7 | 0.204082 | Beta | 0.010412 | 305.5559 | 1191.668 |
| RDV- Day 15 - Baseline Ordinal Score 7 - Ordinal Score 1-3 | 0.122137 | Beta | 0.006232 | 337.1176 | 2423.032 |
| RDV- Day 15 - Baseline Ordinal Score 7 - Ordinal Score 4 | 0.045802 | Beta | 0.002337 | 366.5191 | 7635.814 |
| RDV- Day 15 - Baseline Ordinal Score 7 - Ordinal Score 5 | 0.21374 | Beta | 0.010905 | 301.8357 | 1110.324 |
| RDV- Day 15 - Baseline Ordinal Score 7 - Ordinal Score 6 | 0.076336 | Beta | 0.003895 | 354.7585 | 4292.578 |
| RDV- Day 15 - Baseline Ordinal Score 7 - Ordinal Score 7 | 0.435115 | Beta | 0.0222 | 216.5713 | 281.1627 |
| Comp- Day 15 - Baseline Ordinal Score 7 - Ordinal Score 1-3 | 0.136364 | Beta | 0.006957 | 331.6382 | 2100.375 |
| Comp- Day 15 - Baseline Ordinal Score 7 - Ordinal Score 4 | 0.058442 | Beta | 0.002982 | 361.6506 | 5826.594 |
| Comp- Day 15 - Baseline Ordinal Score 7 - Ordinal Score 5 | 0.149351 | Beta | 0.00762 | 326.6361 | 1860.406 |
| Comp- Day 15 - Baseline Ordinal Score 7 - Ordinal Score 6 | 0.038961 | Beta | 0.001988 | 369.1538 | 9105.793 |
| Comp- Day 15 - Baseline Ordinal Score 7 - Ordinal Score 7 | 0.480519 | Beta | 0.024516 | 199.0831 | 215.225 |
| RDV - HR -Day 29 Mortality - Ordinal Score 1-3 | 1 | Gamma | 0.02551 | 1536.64 | 0.000651 |
| RDV - HR -Day 29 Mortality - Ordinal Score 4 | 0.42 | Gamma | 0.107653 | 15.22109 | 0.027593 |
| RDV - HR -Day 29 Mortality - Ordinal Score 5 | 0.28 | Gamma | 0.047959 | 34.08572 | 0.008215 |
| RDV - HR -Day 29 Mortality - Ordinal Score 6 | 0.82 | Gamma | 0.128061 | 41.00081 | 0.02 |
| RDV - HR -Day 29 Mortality - Ordinal Score 7 | 0.76 | Gamma | 0.113776 | 44.62 | 0.017033 |
| Comp - HR -Day 29 Mortality - Ordinal Score 1-3 | 1 | Gamma | 0.05102 | 384.16 | 0.002603 |
| Comp - HR -Day 29 Mortality - Ordinal Score 4 | 1 | Gamma | 0.036224 | 384.16 | 0.001848 |
| Comp - HR -Day 29 Mortality - Ordinal Score 5 | 1 | Gamma | 0.036224 | 384.16 | 0.001848 |
| Comp - HR -Day 29 Mortality - Ordinal Score 6 | 1 | Gamma | 0.036224 | 384.16 | 0.001848 |
| Comp - HR -Day 29 Mortality - Ordinal Score 7 | 1 | Gamma | 0.05102 | 384.16 | 0.002603 |
| RDV - RR - LoS - Ordinal Score 1-3 | 1.29 | Gamma | 0.096939 | 177.0861 | 0.007285 |
| RDV - RR - LoS - Ordinal Score 4 | 1.29 | Gamma | 0.165816 | 60.52361 | 0.021314 |
| RDV - RR - LoS - Ordinal Score 5 | 1.45 | Gamma | 0.262755 | 30.45325 | 0.047614 |
| RDV - RR - LoS - Ordinal Score 6 | 1.09 | Gamma | 0.221939 | 24.12052 | 0.04519 |
| RDV - RR - LoS - Ordinal Score 7 | 0.98 | Gamma | 0.117347 | 69.74428 | 0.014051 |
| RDV - RR - LoS - Ordinal Score 8 | 1 | Gamma | 0.05102 | 384.16 | 0.002603 |
| Comp - RR - LoS - Ordinal Score 1-3 | 1 | Gamma | 0.05102 | 384.16 | 0.002603 |
| Comp - RR - LoS - Ordinal Score 4 | 1 | Gamma | 0.05102 | 384.16 | 0.002603 |
| Comp - RR - LoS - Ordinal Score 5 | 1 | Gamma | 0.05102 | 384.16 | 0.002603 |
| Comp - RR - LoS - Ordinal Score 6 | 1 | Gamma | 0.05102 | 384.16 | 0.002603 |
| Comp - RR - LoS - Ordinal Score 7 | 1 | Gamma | 0.05102 | 384.16 | 0.002603 |
| Comp - RR - LoS - Ordinal Score 8 | 1 | Gamma | 0.05102 | 384.16 | 0.002603 |
| RDV Rate Ratio - Rehospitalization | 1.666667 | Gamma | 0.085034 | 384.16 | 0.004338 |

FAVI: favipiravir + standard of care; HR: hazard ratio; ICU: Intensive Care Unit; LoS: Length of Stay; MIV: mechanical ventilation; NMA: network meta analysis; OS: Ordinal Score; RDV: remdesivir+standard of care; RR: rate ratio; SoC: standard of care
